# The first complete genome of ‘true’ *Aratinga* genus in comparison to mitogenomes of other parrots from *Arini* tribe

**DOI:** 10.1080/23802359.2016.1250130

**Published:** 2016-11-22

**Authors:** Adam Dawid Urantowka, Tomasz Strzała, Aleksandra Kroczak, Paweł Mackiewicz

**Affiliations:** a Department of Genetics, Wroclaw University of Environmental and Life Sciences, Wroclaw, Poland;; b Department of Genomics, Faculty of Biotechnology, University of Wrocław, Wrocław, Poland

**Keywords:** *Arini*, mitochondrial genomes, parrots, Psittaciformes

## Abstract

*Arini* with 19 genera is the most diversified tribe of the neotropical parrots from *Arinae* subfamily. So far, among this tribe, the genus *Aratinga* appeared to be the most problematic for the taxonomists. The typical representative of this genus is *Aratinga solstitialis*, whose complete mitochondrial genome were sequenced and compared with five other representatives of *Arini* mitogenomes. Despite the conservatism in their general organization, some changes in A + T% composition of individual genes, start/stop codon usage and intergenic regions accumulated during evolution.

All neotropical parrots are classified into *Arinae* subfamily, which is divided into four tribes (Joseph et al. 2012; Schodde et al. 2013). The most taxon-rich tribe *Arini* includes morphologically diverse *Aratinga* genus, which is the most taxonomically controversial. After revision, it was split into: *Eupsittula*, *Psittacara*, *Thectocercus,* and ‘true’ *Aratinga* (Remsen et al. 2013; Urantowka et al. 2013). Here, we report the first mitogenome of *Aratinga*, *Aratinga solstitialis* (GenBank accession no. JX441869) and compared it with other *Arini* representatives: *Ara glaucogularis* (Urantowka 2016), *Eupsittula pertinax* (Pacheco et al. 2011), *Psittacara mitratus* (Urantowka et al. 2016a), *Pyrrhura rupicola* (Urantowka et al. 2016b), and *Thectocercus acuticaudatus* (Urantowka et al. 2013). A phylogenetic analysis confirmed undoubtedly that the obtained mtDNA belongs to *Aratinga solstitialis* (Figure 1).

**Figure 1.**
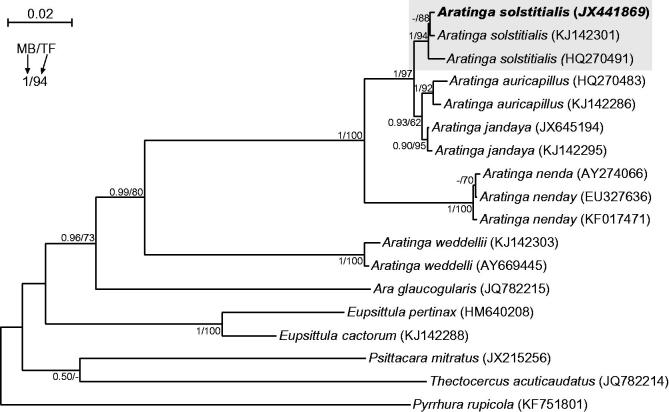
The phylogenetic tree obtained in MrBayes for *nd2* gene indicating that the studied individual (bolded) belongs to *Aratinga solstitialis*. The parrot is kept in culture and its blood sample from which DNA was isolated is available in the laboratory at the Department of Genetics in Wroclaw University of Environmental and Life Sciences under the number ASS16984. Values at nodes, in the order shown, indicate posterior probabilities found in MrBayes (PP) and bootstrap percentages calculated in TreeFinder (BP). In the MrBayes (Ronquist et al. 2012) analysis, separate mixed substitution models were assumed for three codon positions with information about heterogeneity rate across sites as proposed by PartitionFinder (Lanfear et al. 2012). We applied two independent runs, each using four Markov chains. Trees were sampled every 100 generations for 10,000,000 generations. After obtaining the convergence, trees from the last 2,447,000 generations were collected to compute the posterior consensus. In the case of TreeFinder (Jobb et al. 2004), the separate substitution models were selected for three codon positions according to Propose Model module in this program, and 1000 replicates were assumed in the bootstrap analysis. The posterior probabilities <0.5 and bootstrap percentages <50 were omitted or marked by a dash ‘-’.

The length of *Aratinga solstitialis* genome (16,984 bp) is identical with *Psittacara mitratus*. *Thectocercus acuticaudatus* has the longest of the studied genomes (16,998 bp) and *Eupsittula pertinax* the shortest (16,980 bp). All mitogenomes revealed the same gene composition, order and orientation. The genomic A + T% of studied *Arini* is in the range for other avian mitogenomes (Castellana et al. 2011). *Pyrrhura rupicola* genome has the lowest A + T content (52.5%), whereas *Aratinga solstitialis* has the highest (54.1%). The greatest difference between the maximum and minimum of A + T% was found for tRNA-Tyr gene (7.1%) and the lowest for 12srRNA (0.7%).

The mean percent distance between the genomic sequences is 10.36. The largest difference to others shows *Pyrrhura rupicola* (about 11%), whereas the closest are pairs *Psittacara mitratus* – *Thectocercus acuticaudatus* and *Aratinga solstitialis* – *Ara glaucogularis* with 9.7% difference.

Several differences are present in start/stop codons of protein coding genes. Gene *nd2* from *Eupsittula pertinax* uses ATG as start codon, whereas others use ATA. Stop codons of *atp8* and *nd4L* genes from this species end with the truncated TA_ codons instead of the complete TAA in other investigated *Arini*. The complete TAA stop codon is also present in *Psittacara mitratus* and *Thectocercus acuticaudatus atp6* gene, while in other genera this codon is incomplete (TA_). Only *Aratinga solstitialis* and *Ara glaucogularis nd5* genes end with TAG codon, in contrast to others, which have TAA stop codon.

One extra (not translated) nucleotide, which has been reported for reptile and avian species (Mindell et al. 1998; Russell & Beckenbach 2008), was also found for all six analysed *nd3* genes. However, instead of additional adenine, cytosine was detected in these genes from studied *Ara*, *Aratinga*, *Psittacara*, *Pyrrhura,* and *Thectocercus*, whereas thymine in *Eupsittula pertinax*.

The length of control region (CR) in *Aratinga solstitialis* (1482 bp) is the same as in *Thectocercus acuticaudatus*. The shortest CR was found in *Psittacara mitratus* (1458 bp) and the longest in *Pyrrhura rupicola* (1492 bp). Small insertion or deletion led to variation in the length of eight intergenic regions (up to 19 bp), as well as two rRNAs (up to 10 bp) and seven tRNAs (up to 4 bp). The tRNA-Val/16S rRNA spacer was identified only in *Ara glaucogularis* and the *atp6*/*cox3* overlap in *Psittacara mitratus* and *Thectocercus acuticaudatus*.
